# Impact of thyroid volume on serum ionized calcium and PTH levels after total thyroidectomy

**DOI:** 10.1590/1516-3180.2024.0441.13062025

**Published:** 2025-11-21

**Authors:** Nicolas Johnson de Oliveira Vieira, Lucas Norambuena Aulicino, João Victor Marques Cruz Helene de Oliveira, Inês Nobuko Nishimoto, Rogério Aparecido Dedivitis

**Affiliations:** I Past-undergraduate student, Faculdade de Ciências Médicas de Santos, Centro Universitário Lusíada, Santos (SP), Brazil.; II Past-undergraduate student, Faculdade de Ciências Médicas de Santos, Centro Universitário Lusíada, Santos (SP), Brazil.; III Past-undergraduate student, Faculdade de Ciências Médicas de Santos, Centro Universitário Lusíada, Santos (SP), Brazil.; IV Assistant Professor, Faculdade de Ciências Médicas de Santos, Centro Universitário Lusíada, Santos (SP), Brazil.; V Full Professor, Department of Surgery, Faculdade de Ciências Médicas de Santos, Centro Universitário Lusíada, Santos (SP), Brazil.

**Keywords:** Hypoparathyroidism, Thyroidectomy, Complications, postoperative, Parathyroid hormone, Hypocalcemia, Parathyroid, Thyroid surgery, Hypocalcemia, PTH, Calcium level

## Abstract

**BACKGROUND:**

The relationship between thyroid gland volume and hypoparathyroidism after total thyroidectomy remains controversial.

**OBJECTIVE:**

To evaluate thyroid gland mass as a risk factor for hypoparathyroidism after total thyroidectomy.

**DESIGN AND SETTING:**

A Retrospective cross-sectional observational study was conducted at Centro Universitário Lusíada (UNILUS), Santos/SP, Brazil.

**METHODS:**

Patients undergoing total thyroidectomy between January 2022 and September 2023 were retrospectively evaluated for serum levels of ionized calcium and ultrasensitive parathyroid hormone (PTH), measured preoperatively and 30–60 days postoperatively, and thyroid mass, obtained by weighing the specimen.

**RESULTS:**

A total of 174 patients were evaluated, with a predominance of women (89.7%), a median age of 50.5 years, and a median goiter volume of 36.3 mL. A reduction in both PTH and ionized calcium levels was observed in the postoperative period compared with the preoperative period (P < 0.05). No significant changes were observed in PTH levels and volume (P = 0.481). For calcium, there was a tendency towards an association between volume measurements and its change between the pre- and postoperative periods, which was marginally significant (P = 0.051).

**CONCLUSION:**

There was a marginal association between volume and changes in pre- and postoperative ionized calcium levels, but no significant association with pre- and postoperative PTH measurements.

## INTRODUCTION

 Total thyroidectomy is the most common endocrine surgery. Postoperative hypocalcemia is the most common complication of total thyroidectomy. Its incidence varies from 30% to 60%, and most patients recover completely. It is not always associated with accompanying symptoms and, in most cases, resolves in less than 6 months. The incidence of transient and permanent hypocalcemia varies from 19% to 38% and 0% to 3%, respectively^1^. 

 The mechanisms include direct injury, devascularization, venous drainage obstruction, or inadvertent excision of the parathyroid glands. A systematic review identified predictors of transient and permanent hypocalcemia after total thyroidectomy. Independent clinical predictors of permanent hypocalcemia included reoperation for bleeding, identification of fewer than two parathyroid glands, Graves’ disease, and larger thyroid volume on multivariate analysis^2^. However, in another meta-analysis, the significant predictors of transient hypocalcemia were younger age, female sex, parathyroid autotransplantation, inadvertent parathyroid excision, Graves’ disease, thyroid cancer, central compartment clearance, severe preoperative vitamin D deficiency, and low postoperative 24-hour parathyroid hormone (PTH) levels^3^. 

 However, the effect of thyroid gland volume as a risk factor for hypoparathyroidism remains controversial. In a prospective review of 121 patients undergoing total thyroidectomy, the thyroid volume was calculated preoperatively using ultrasound and by weighing the surgical specimen. When analyzing the incidence of complications based on the quartiles of gland weight and volume, no significant difference was observed in the incidence of transient or permanent hypoparathyroidism^4^. 

## OBJECTIVES

 This study aimed to evaluate thyroid gland mass as a risk factor for decreased ionized calcium and PTH levels following total thyroidectomy. 

## METHODS

 This study was approved by the Institutional Review Board of Fundação Lusíada, Centro Universitário Lusíada (UNILUS) under number 857/2023, on August 4th, 2023. All patients who underwent total thyroidectomy between January 2022 and September 2023 were retrospectively evaluated by reviewing their medical records. The inclusion criteria were as follows: patients aged > 18 years who underwent total thyroidectomy. Exclusion criteria included any type of cervical clearance, altered calcium homeostasis, prior surgical or radiotherapy treatment in the cervical region, and incomplete medical records. 

 All patients underwent surgery by the same surgical team using a standardized technique. The following aspects were evaluated: biochemical monitoring of mineral homeostasis by measuring ionized calcium and ultrasensitive PTH levels, assessed preoperatively and between 30 and 60 days postoperatively, and the mass of the thyroid gland obtained by weighing the specimen after removal. 

 Statistical analyses were performed as follows: frequency distribution was used to describe categorical variables, and measures of central tendency (mean and median) and variability (range and standard deviation) were used for numerical variables. The Wilcoxon signed-rank test was used to assess the association between preoperative and postoperative measurements of numerical variables (PTH and ionized calcium). We calculated the difference between postoperative and preoperative measurements of ionized calcium and PTH levels, creating a categorical variable (increase or decrease), and the association between the numerical variable and this new categorical variable was evaluated using the non-parametric Mann–Whitney U test. The Shapiro-Wilk test was used to check the normality of the numerical data for variables (PTH, ionized calcium, and volume). A significant level of 5% was used for all statistical tests. The statistical software STATA version 18 (StataCorp LLC, College Station, Texas, United States) was used for all the statistical analyses^5^. 

## RESULTS

 A total of 174 patients were evaluated, with a predominance of women (89.7%), a median age of 50.5 years, and a median goiter volume of 36.3 mL (**Table 1**). 

**Table 1:** Distribution of the study population according to demographic and clinical variables (n = 174)

**Variable**	**Category / Measures**	**Freq. (%) / Measures**
Gender	Female	156 (89,7)
	Male	18 (10,3)
Age	Range	18 – 83
	Median	50,5
	Mean (SD)	50,8 (13,8)
Volume (cm)	Range	4,7 – 495
	Median	36,3
	Mean(SD)	49,0 (49,4)

SD = standard deviation.

 When comparing preoperative and postoperative PTH measurements, it was noted that the postoperative measurements were lower, and this difference was statistically significant (P < 0.001). The same was observed for ionized calcium; although there was a slight reduction, the difference was statistically significant (P = 0.0004) (**Table 2**). 

**Table 2:** Association between preoperative and postoperative measurements of parathyroid hormone and ionized calcium (n = 174)

**Variable**	**Measures**	**Pré**	**Post**	**P value**
PTH	Range	15 – 105	5,1 – 85	< 0,001
	Median	44,3	39,6	
	Mean (SD)	46,3 (17,8)	41,1 (14,9)	
Ionized Calcium	Range	1,00 – 1,35	0,88 – 1,38	0,0004
	Median	1,23	1,22	
	Mean (SD)	1,23 (0,05)	1,22 (0,07)	

PTH = parathyroid hormone; SD = standard deviation; P value obtained using the paired Wilcoxon signed-rank test.

 To assess the association between preoperative and postoperative measurements in relation to volume, we created a difference variable by subtracting preoperative measurements from postoperative measurements (**Table 3**). 

**Table 3:** Distribution of differences between postoperative and preoperative parathyroid hormone and ionized calcium levels

**Variable**	**Measures**	**Measures**		**Difference (Post- minus pre-)**
		**Pre**	**Post**	
PTH	Range	15 – 105	5,1 – 85	(-73,0) – 35,2
	Median	44,3	39,6	(-3,0)
	Mean (SD)	46,3 (17,8)	41,1 (14,9)	(-5,2) (13,5)
Ionized Calcium	Range	1,00 – 1,35	0,88 – 1,38	(-0,41) – 0,34
	Median	1,23	1,22	(-0,02)
	Mean (SD)	1,23 (0,05)	1,22 (0,07)	(-0,015) (0,072)

PTH = parathyroid hormone; SD = standard deviation.

 An increase was considered when the difference was positive, that is, the postoperative measurement was greater than the preoperative measurement, and a decrease was considered when the difference was negative, or the postoperative measurement was lower than the preoperative measurement. The volumes were then compared in relation to the increase or decrease in pre- and postoperative values. It is observed that the volumes were larger when the postoperative PTH difference was greater than the preoperative, with a median volume of 37.7 cm compared to a median volume of 35.1 cm when the postoperative PTH was lower than the preoperative, but no significant difference was observed (P = 0.481) (**Table 4**). 

**Table 4:** Association between volume and differences in parathyroid hormone measurements (difference = postminus pre)

**Variable**	**Measures**	**Difference in PTH (Post-Pre)**		**P value**
		**Reduction**	**Increase**	
Volume (cm)	n	112	62	0,481
	Range	4,7 – 280,0	9,3 – 495,0	
	Median	35,1	37,7	
	Mean (SD)	46,2 (37,8)	54,0 (65,4)	

SD = standard deviation; PTH = parathyroid hormone; P value obtained using the Mann-Whitney U test.

 The volumes were larger when the postoperative calcium difference was greater than the preoperative value, with a median volume of 40.4 mL compared with a median volume of 31.3 mL when the postoperative ionized calcium was lower than the preoperative value. However, a tendency towards an association was observed between the volume and ionized calcium (post- and preoperative), which was marginally significant (P = 0.051) (**Table 5**). 

**Table 5:** Association between volume and differences in ionized calcium measurements (difference = post minus preoperative), excluding one case in which the postoperative measurement was equal to the preoperative measurement (n = 173)

**Variable**	**Measures**	**Difference in ionized calcium (Post- minus pre-)**		**P value**
		**Reduction**	**Increase**	
Volume (cm)	n	102	71	0,051
	Range	9 – 126,2	4,7 – 495	
	Median	31,3	40,4	
	Mean (SD)	42,0 (28,1)	59,6 (68,4)	

SD = standard deviation; P value obtained using the Mann-Whitney U test.

 Therefore, it was found that the preoperative and postoperative measurements were different, with a decrease in the postoperative values compared to the preoperative values for both PTH and ionized calcium (P < 0.05). However, no significant changes were observed in the increase or decrease in PTH levels or volume (P > 0.05). For calcium, there was a tendency towards an association between volume measurements and the decrease or increase between the preoperative and postoperative periods, which was marginally significant (P = 0.051). 

## DISCUSSION

 Total thyroidectomy can lead to hypoparathyroidism, the most frequent complication, with an incidence of permanent hypoparathyroidism of 4.11% at 6 months postoperatively.^6^ Low postoperative levels of PTH and the resulting hypocalcemia may be associated with the accidental removal of one or more glands or compromised blood supply. Postoperative hypocalcemia can present with serious complications and cause significant morbidity.^7^

 Monitoring postoperative PTH and serum calcium levels is the best predictor for identifying hypoparathyroidism and treating the resulting symptom, hypocalcemia; however, there is no consensus on the timing, patient selection, and cutoff points for PTH levels.^7^ A meta-analysis included 23 studies. Twelve significant risk factors for postoperative hypocalcemia were identified: hypoparathyroidism, OR = 5.58; total thyroidectomy, OR = 3.59; hypomagnesemia, OR = 2.85; preoperative vitamin D deficiency, OR = 2.32; female gender, OR = 1.49; thyroid malignancy, OR = 1.85; thyroiditis, OR = 1.48; sub-sternal multinodular goiter, OR = 1.70; parathyroidectomy, OR = 1.58; central compartment neck dissection, OR = 1.17; modified radical neck dissection, OR = 1.57; and central neck dissection, OR = 1.54.^8^ Another metanalysis showed significant predictors of transient hypocalcemia: younger age, female gender, parathyroid autotransplantation, inadvertent parathyroid excision, Graves’ disease, thyroid cancer, central compartment dissection, preoperative vitamin D deficiency, and low PTH levels 24 hours postoperatively.^3^ None of these studies considered gland volume as a possible risk factor. 

 A total of 2,937 patients were evaluated for hypoparathyroidism. The rates of transient and permanent hypoparathyroidism were 25.20% and 2.69%, respectively. A large thyroid mass was designated in cases in which the gland volume represented a difficulty that interfered with the regular progress of the procedure. This was a subjective criterion based on the surgeon’s perception and experience. The occurrence of transient hypoparathyroidism was independently linked to thyroid weight (P < 0.001).^9^ In a group of 227 patients, 74 (32.6%) had goiters with a weight exceeding 250 g (massive goiter), and 153 (67.4%) had masses between 100 g and 250 g. Patients with massive goiters had higher rates of transient hypoparathyroidism (41.9% vs. 25.5%).^10^

 Thyroid volume was calculated preoperatively using ultrasonography and, together with the final specimen weight, correlated with the development of postoperative complications in 131 patients undergoing total thyroidectomy. When analyzing the incidence of complications based on the quartiles of weight and glandular volume, no significant differences were observed in the incidence of transient or permanent hypoparathyroidism in any of the groups. No fewer parathyroid glands were visualized intraoperatively in patients with larger thyroid glands, nor was there an increased number of glands accidentally removed during surgery. In fact, a certain protective trend was observed regarding the number of visualized glands and gland size, and the correlation between thyroid volume and accidental gland removal, with no significant differences.^4^

 There is controversy in the literature regarding the possible influence of thyroid gland masses on hypoparathyroidism after total thyroidectomy. In our study, the operation led to a decrease in ionized calcium levels, but no significant association was observed with the mass. Regarding PTH levels, the observed decrease in the PTH levels was not statistically significant. 

## CONCLUSION

 There may have been an association between volume and changes in preoperative and postoperative ionized calcium measurements, although the obtained value (P = 0.051) was considered marginally significant. No significant association was observed between pre- and postoperative PTH measurements. 
